# Evaluation of breast stiffness measured by ultrasound and breast density measured by MRI using a prone-supine deformation model

**DOI:** 10.1186/s40364-019-0171-1

**Published:** 2019-09-11

**Authors:** Jeon-Hor Chen, Siwa Chan, Yang Zhang, Shunshan Li, Ruey-Feng Chang, Min-Ying Su

**Affiliations:** 1 John Tu and Thomas Yuen Center for Functional Onco-Imaging, University of California, 164 Irvine Hall, Irvine, CA 92697-5020 USA; 2 Department of Radiology, E-Da Hospital and I-Shou University, Kaohsiung, Taiwan; 3 Graduate Institute of Biomedical Electronics and Bioinformatics, National Taiwan University, Taipei, Taiwan; 4 Department of Radiology, Tzu-Chi General Hospital, Taichung, Taiwan

**Keywords:** Breast stiffness, Breast density, Ultrasound elastography, Magnetic resonance imaging, Finite element model

## Abstract

**Background:**

This study evaluated breast tissue stiffness measured by ultrasound elastography and the percent breast density measured by magnetic resonance imaging to understand their relationship.

**Methods:**

Magnetic resonance imaging and whole breast ultrasound were performed in 20 patients with suspicious lesions. Only the contralateral normal breasts were analyzed. Breast tissue stiffness was measured from the echogenic homogeneous fibroglandular tissues in the central breast area underneath the nipple. An automatic, computer algorithm-based, segmentation method was used to segment the whole breast and fibroglandular tissues on three dimensional magnetic resonanceimaging. A finite element model was applied to deform the prone magnetic resonance imaging to match the supine ultrasound images, by using the inversed gravity loaded transformation. After deformation, the tissue level used in ultrasound elastography measurement could be estimated on the deformed supine magnetic resonance imaging to measure the breast density in the corresponding tissue region.

**Results:**

The mean breast tissue stiffness was 2.3 ± 0.8 m/s. The stiffness was not correlated with age (*r* = 0.29). Overall, there was no positive correlation between breast stiffness and breast volume (*r* = − 0.14), or the whole breast percent density (*r* = − 0.09). There was also no correlation between breast stiffness and the local percent density measured from the corresponding region (*r* = − 0.12).

**Conclusions:**

The lack of correlation between breast stiffness measured by ultrasound and the whole breast or local percent density measured by magnetic resonance imaging suggests that breast stiffness is not solely related to the amount of fibroglandular tissue. Further studies are needed to investigate whether they are dependent or independent cancer risk factors.

## Introduction

Breast connective and epithelial tissues attenuate x-ray more than fat and thus show higher signal intensity than fat on mammography, an appearance that is referred to as “mammographic densities.” Mammographic density (MD) is a well-documented risk factor for developing breast cancer [1]. Histological studies have shown that high MD tissue had a significantly greater proportion of stroma, collagen, epithelium, and increased proteoglycan expression, compared to low MD tissue [1–3], but how they are related to cancer risk is not clear.

Breast stiffness, reflecting the physical forces generated by interactions between cells, and between cells and the extracellular matrix, influences a variety of cell functions including cell growth, survival, motility and differentiation [4]. Kass et al. suggested that the mechanical properties of the tissue might also influence breast cancer risk [5]. In normal mammary epithelial cells, increasing extracellular matrix (ECM) stiffness alone induces malignant phenotypes [6]. A recent study [7] noted that an estimate of breast tissue stiffness, derived from measurements of the volume and the projected area of the compressed breast during mammography, was associated with breast cancer risk and could be used to improve risk prediction. Molecules that mediate the influence of the ECM and the stiffness of stroma, and how they are associated with breast cancer, are being investigated [8, 9].

Tissue stiffness can be evaluated by ultrasound (US) elastography methods. Currently two types of US elastography are available: strain elastography and shear wave elastography (SWE) [10]. Strain elastography is based on the comparison of echo signals acquired before and after compression of the tissue. The results are displayed as an elastographic image, which shows the relative stiffness of the tissues [11, 12]. In contrast, SWE, including the acoustic radiation force impulse imaging (ARFI) and the supersonic shear-wave imaging, can provide a quantitative assessment of stiffness by measuring the propagation speed of shear waves generated by the acoustic radiation force [13].

In recent years, US elastography has been used for the clinical diagnosis and evaluation of breast tumors. It has been shown that breast cancer is characterized by increasing stiffness, and malignant breast lesions exhibited high stiffness not only in the lesion but also in the surrounding tissue, whereas benign lesions demonstrated low stiffness in both lesion and the surrounding area [14, 15]. The content of collagen fiber of malignant lesions was significantly higher than that of benign lesions [16]. US elastography of normal breast tissue is less studied. In a study [17] of normal breast tissue stiffness using a new SWE technique, i.e. virtual touch tissue imaging quantification (VTIQ), in 132 breasts, the tissue stiffness of the breast parenchyma was significantly higher compared to that of fatty tissue.

Since both high MD and stiffness are associated with increased breast cancer risk, it is of interest to investigate if a direct link exists between high breast density and high tissue stiffness. The purpose of this study was to compare tissue stiffness measured from the dense area in US image with magnetic resonance (MR) measurement of the whole breast volumetric density and the local density from the region where the US stiffness was measured. In this study we did not measure MD for the correlation was because MD is a two dimensional image, which suffers from the problem of tissue overlapping, thus cannot accurately measure breast density. MR-measured breast density, on the other hand, is three dimensional and has clear fat-fibroglandular tissue contrast. The association between density and stiffness was investigated.

## Materials and methods

### Subjects

In a period of 1 month, twenty women (age range 24–78, mean 51.7 y/o) with suspicious unilateral breast lesion were enrolled. The subject received routine US of the bilateral breasts and breast lesions in the unilateral breast were confirmed. US elastography of the normal breast side was then performed. On the same day, several hours after the US studies, breast MRI of the bilateral breasts was acquired. Of these 20 subjects, 18 were subsequently confirmed to have unilateral breast cancer and two subjects had benign lesions. In this study, only the contralateral normal breast tissue was analyzed and correlated between US and MRI.

### Ultrasound Elastography

US elastography of the breast was acquired with a Siemens ACUSON S2000™ ultrasound system, an automated breast volume scanner. The system adopted a new SWE technique VTIQ, which was based on an ARFI technology. VTIQ uses an acoustic push pulse followed by detection pulses to calculate shear wave speed in unit of (m/s). In general, shear wave speed increases with tissue stiffness, expressed in Shear Wave propagation velocity or deduced Young Modulus in kilopascals (kPa) or meters per second (m/s). The US elastography measurement procedures were: 1) anatomical locations for measurement defined by region of interest (ROI) were selected; 2) acoustic push pulse was applied adjacent to region of interest (ROI); 3) tracking beams (sensitive to greater than 1/100 the wavelength of sound) were applied adjacent to the acoustic push pulse; and 4) the time between the generation of the shear wave and the passing of shear wave peak at an adjacent location was utilized to compute the shear wave velocity. Since most dense tissues are in the “central” breast area below the nipple, the ROI for the shear force measurement was placed here. The mean depth of the ROI from the skin surface was 1.41 ± 0.38 cm (range 0.6 cm ~ 2.2 cm).

### MR imaging and breast segmentation

The breast MR images (MRI) were acquired with a 1.5 Tesla Siemens scanner (Aera; Siemens Healthcare, Erlangen, Germany) by using a 16-channel phased-array coil. The breast volume and density were measured on pre-contrast spin echo T1W images (TR, 726 msec; TE, 8.2 msec; field of view, 320 mm; slide thickness, 3 mm) by using a template-based automatic segmentation method, details published before [18]. With this method, the chest body region on a middle slice was used as the template. The chest template was mapped to each subject’s image space to obtain a subject-specific chest model for exclusion. The chest and muscle boundaries determined on the middle slice were used as the reference for the segmentation of adjacent slices, and the process continued superiorly and inferiorly until all 3D slices were segmented. After the breast was segmented, the bias-field correction and fuzzy-C-means algorithm was applied to separate the fat from the fibroglandular tissue [19]. In this study, only the normal breast of the women was analyzed.

### Deformation of segmented breast MR images from prone to supine position

Because the breast MRI was acquired in a prone position (facing down) and the ultrasound was acquired in a supine position (facing up), a deformation processing was performed to change the MR images from prone to supine, so that the measured regional breast density on MRI could be correlated with stiffness measured by US. The deformation was performed using the finite element model (FEM) based biomechanical simulation [20]. The FEM was generated by using the meshing package TetGen [21], with a marching cube algorithm [22, 23]. The breast was meshed into a large number of tetrahedrons that have various sizes. In this way, the geometry of the breast could be modified but the topology could be kept [20, 24]. Each tetrahedron was modeled as isotropic and homogenous element, which represented one type of tissue, either fat or fibroglandular tissue.

**Fig. 1:**
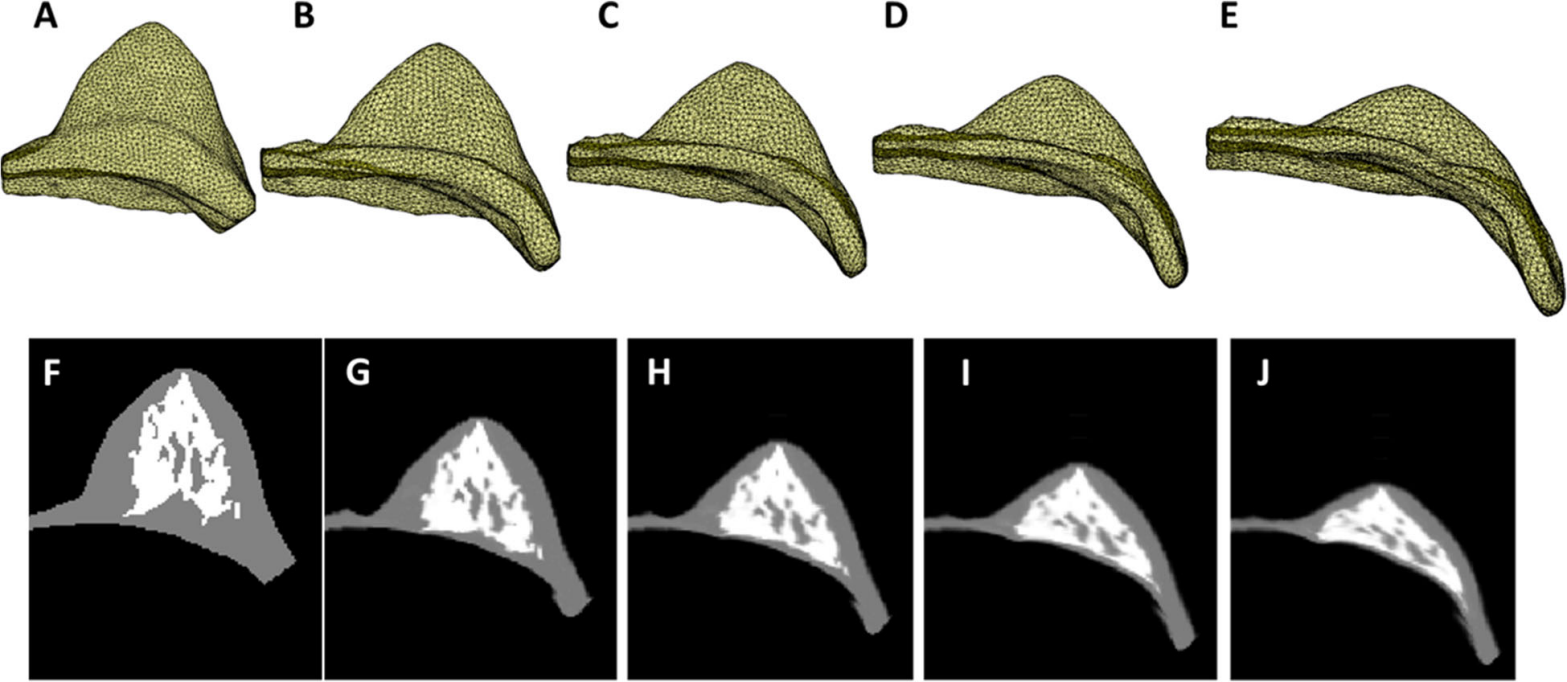
The iterative process to deform the prone MR images to simulate the supine images. **a** the 3D FEM model constructed from the MR segmentation results. **b** the intermediate state between the original and the zero-gravity state. **c** the zero-gravity state. **d**: the intermediate state between the zero-gravity state and the inversed gravity loaded state. **e** the final inversed gravity loaded state. **f**-**j** the middle slice of 3D model corresponding to the states shown in (**a**-**e**)

**Fig. 2:**
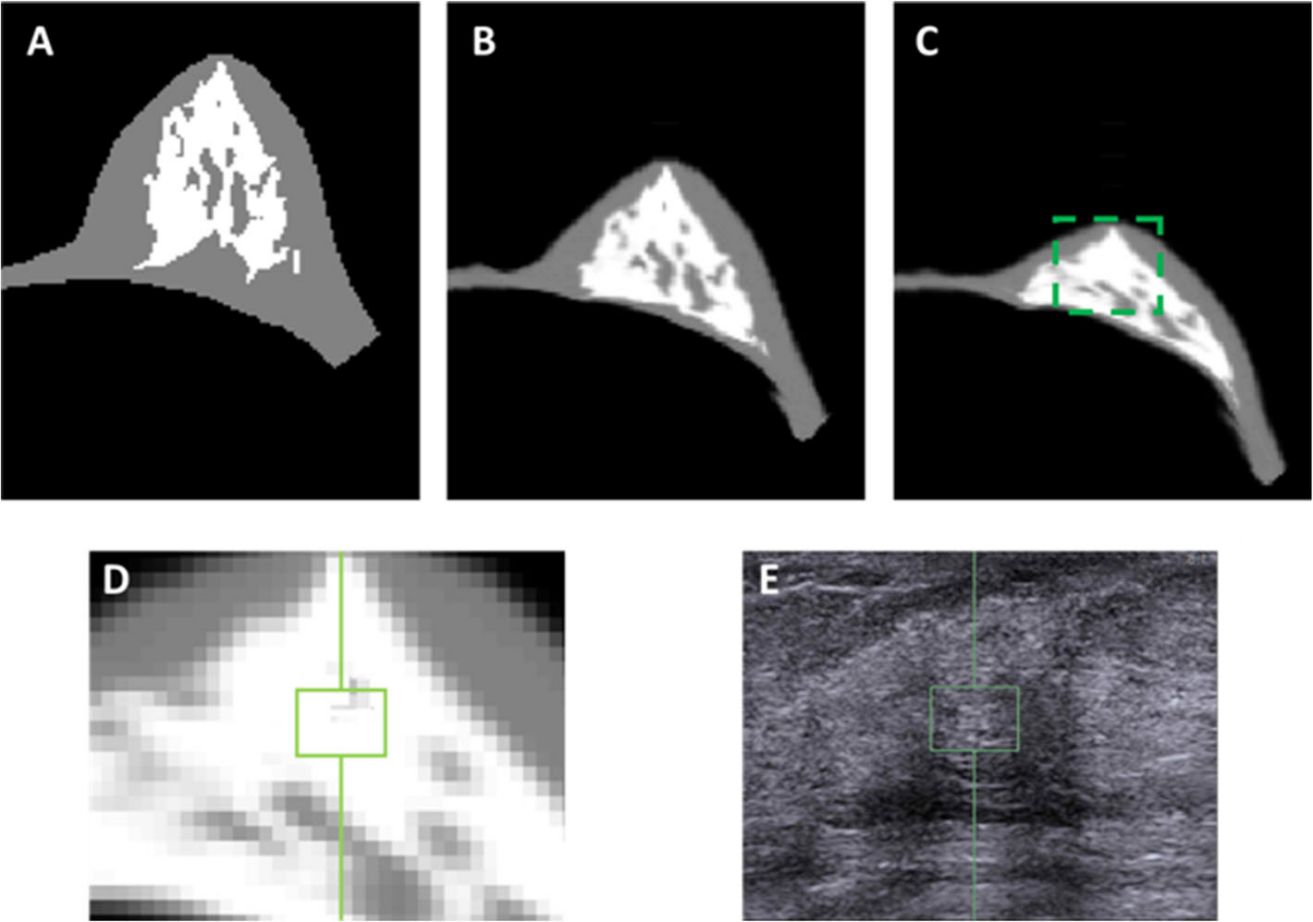
The deformation of MRI to match the US images in left breast of a 48-year-old woman for locating the corresponding stiffness ROI. **a** The middle slice of the original model constructed from the MR segmentation results. **b** The middle slice of the zero-gravity state. **c** The middle slice of the final inversed gravity loaded state. The box is corresponding to the ultrasound image. **d** The zoomed-in area to show the corresponding stiffness measurement ROI in the ultrasound elastography. **e** The ultrasound image with the selected ROI used for stiffness measurement

**Fig. 3:**
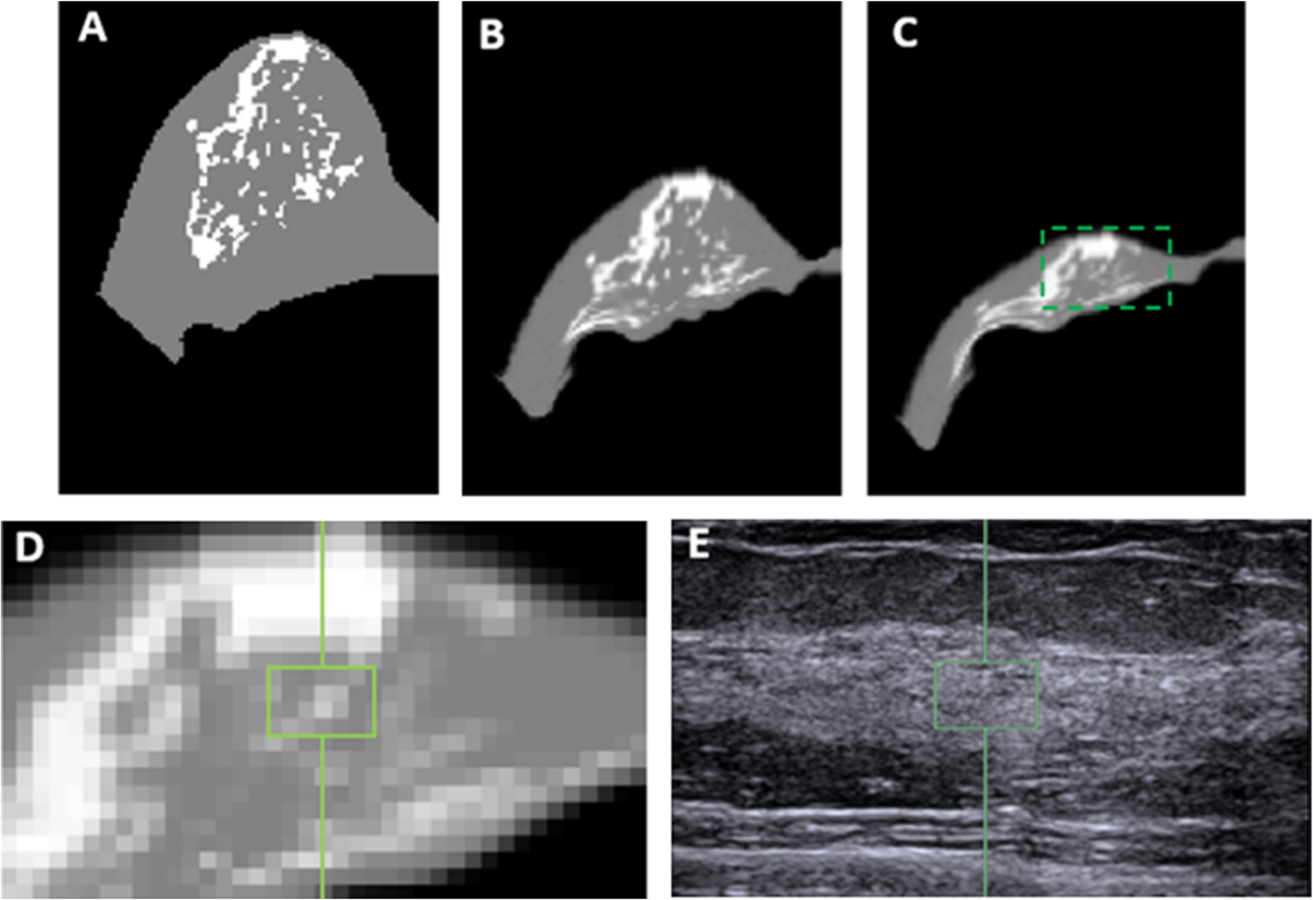
The deformation of MRI to match the US images in right breast of a 42-year-old woman for locating the corresponding stiffness ROI. **a** The middle slice of the original model constructed from the MR segmentation results. **b** The middle slice of the zero-gravity state. **c** The middle slice of the final inversed gravity loaded state. The box is corresponding to the ultrasound image. **d** The zoomed-in area to show the corresponding stiffness measurement ROI in the ultrasound elastography. **e** The ultrasound image with the selected ROI used for stiffness measurement

The neo-Hookean nonlinear material model was utilized to simulate the zero-gravity state of the breast [25]. The bulk moduli of the fatty and the fibroglandular tissues were determined as 3400 Pa and 50,000 Pa, respectively [26]. An iterative scheme was applied, and the posterior boundary of the breast adjacent to the chest wall was fixed as the boundary conditions. The simulation was performed with the open source package NiftySim [27], which utilizes a Total Lagrangian Explicit Dynamic (TLED) algorithm [20, 28]. Figure 1 shows different stages of the inversed gravity loaded transformation. The iteration stops when the depth of the deformed breast in MRI was equal to the depth of the breast in ultrasound image. Then an ROI corresponding to the US elastography measurement window (depth from skin and the size) was placed on the central slice that contained the nipple. The percent breast density was calculated by dividing the fibroglandular tissue area inside the ROI box by the area of the whole box. Figures 2 and 3 show two case examples of women with high and low breast density, respectively. They illustrate the deformation of MR images to simulate the US images, to find the corresponding tissue region for measurement of local breast density.

### Statistics

The Pearson’s correlation was applied to assess the association between US-measured breast stiffness and patients’ age, breast volume, whole breast percent density, and local percent density. Correlation coefficient r was used to indicate the degree of correlation between two parameters. No correlation was defined when *r* < 0.3. A weak correlation was defined when *r* ≥ 0.3, but less than 0.5. Moderate correlation was when *r* ≥ 0.5, but less than 0.7. A strong correlation was when *r* ≥ 0.7.

## Results

### Measured breast stiffness and breast density

The breast stiffness measured from the most dense retroareolar area in these 20 women ranged from to 1.0 to 4.2 (m/s), with the mean ± STD of 2.3 ± 0.8 (m/s). The breast volume of the 20 women ranged from 320.7cm^3^ to 880.3cm^3^ (mean ± STD, 584.4 ± 170.4 cm^3^), and the fibroglandular tissue volume ranged from 33.4cm^3^ to 161.4cm^3^ (mean ± STD, 72.7 ± 31.3 cm^3^). The percent breast density of the 20 women ranged from 5.0 to 28.4% (mean ± STD, 13.1 ± 5.8%).

### Reproducibility of breast stiffness measurement

To determine the reproducibility of the breast stiffness measures using US, the experienced US technician selected three to five ROIs at the different locations but comparable depth of the breast which showed dense tissue. In most of the patients, three locations were measured (on average, 1.41 cm vs. 1.44 cm vs. 1.48 cm). The coefficient of variation (CV) was used to analyze the consistency of the multiple measurements of the breast stiffness at different locations. On average, the CV across the 20 subjects was 12.7% (range 0.7–41.5%). Nine patients had CV below 10%, eight patients showed CV between 10 and 20%, and three patients had CV more than 20%. In this study, only the breast stiffness measured from the ROI showing the most dense breast tissue area in US was used to correlate with the breast density measurement from MRI.

### Correlation of breast stiffness with whole breast density

**Fig. 4:**
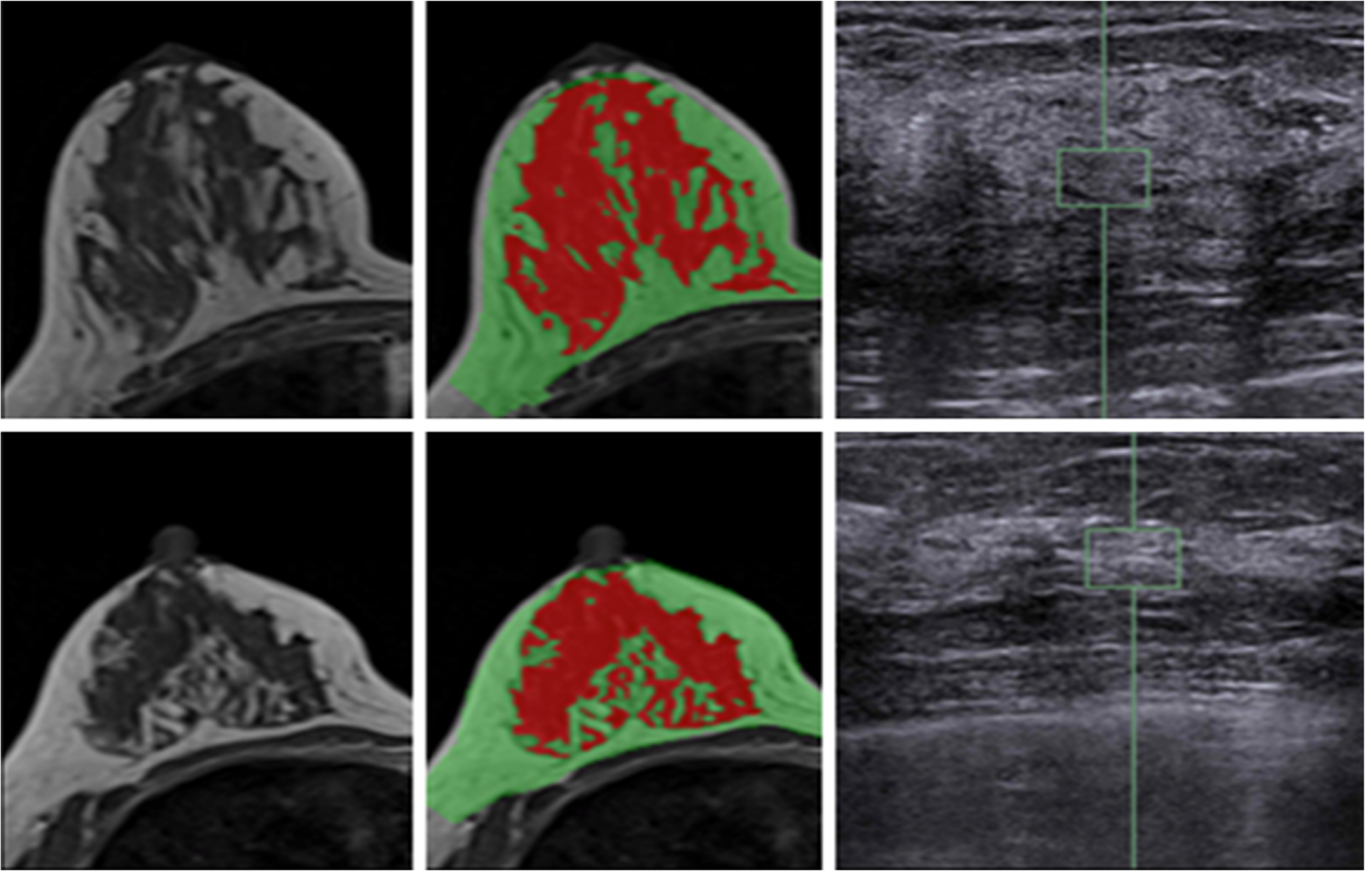
Two case examples illustrating discordant results between density and stiffness. The original MRI (left panel), segmented breast density (middle panel) and the stiffness ROI placed on ultrasound image (right panel) are shown. The upper Case #1 (46 y/o): breast volume 725 cm^3^, whole breast density 22.3%, local breast density 51.0%, and breast stiffness 1.45 m/s. The lower Case #2 (53 y/o): breast volume 411 cm^3^, whole breast density 14.1%, local breast density 16.4%, and breast stiffness 3.92 m/s. Case #1 has higher density but lower stiffness

**Fig. 5:**
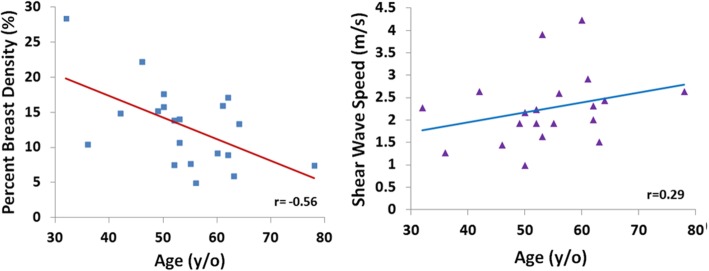
The correlation of whole breast percent density measured on MRI with age (left), and the stiffness measured by US elastography with age (right)

**Fig. 6:**
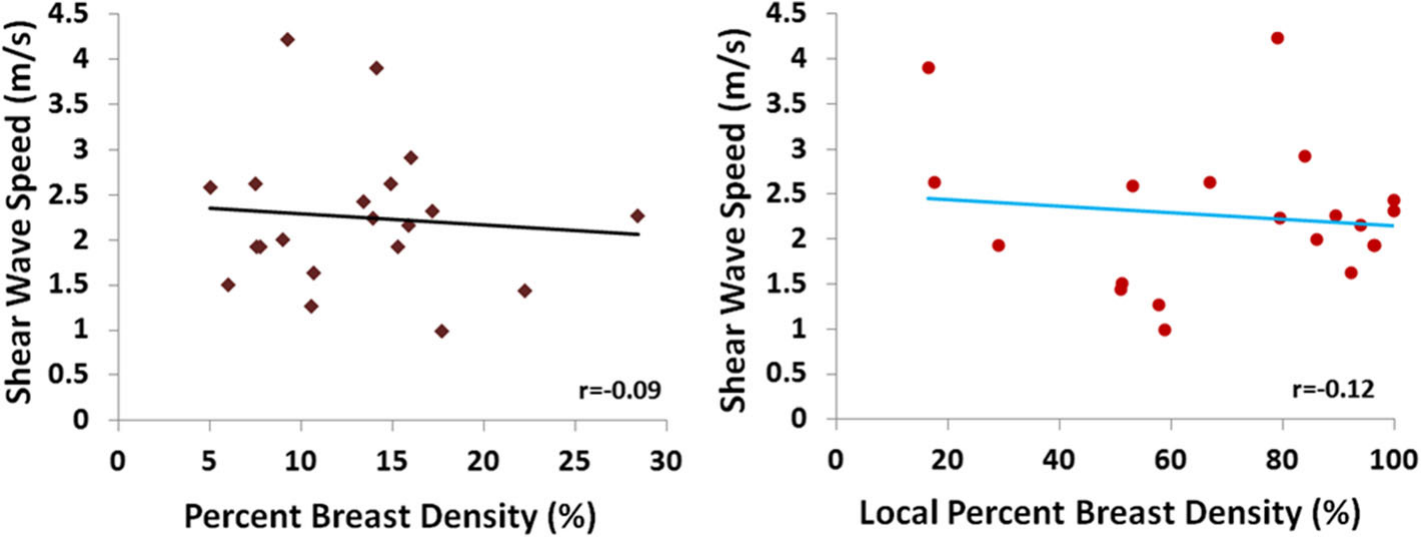
There is no correlation between dense tissue stiffness measured on US and the whole breast percent density measured on MRI (left), or the local percent density measured on MRI from the US-defined ROI (right)

Figure 4 shows two case examples. It can be seen that the ROI used to measure stiffness are selected from the homogeneous dense tissue areas appeared on ultrasound images. In Fig. 4, Case#1 has a higher breast volume (725 cm^3^), whole breast percent density (22.3%), and local percent density (51.0%) compared to Case#2 (411 cm^3^, 14.1, and 16.4%, respectively). However, the breast stiffness is lower in Case#1 compared to Case#2 (1.4 vs. 3.9 m/s). Figure 5 shows the association of the whole breast percent density measured by MRI with the age of the subjects, and as expected a clear trend of decreasing density with age was noted (*r* = − 0.56). On the other hand the breast stiffness was not correlated with age (*r* = 0.29). Overall, there was no correlation between breast stiffness and breast volume (*r* = − 0.14). Also, Fig. 6 shows that there was no correlation between breast US-stiffness and whole breast percent density (r = − 0.09) or local density (*r* = − 0.12) measured by MRI.

## Discussion

In this study we measured the stiffness of the normal dense breast tissue by US elastography and the percent breast density measured by MR imaging. The innovation was that the US elastography was measured by using ACUSON S2000™ ultrasound system, an automated breast volume scanner, and as such the different levels of breast tissues from a large field of view were seen on the US image, which could be used to estimate and correspond with tissues seen on the deformed MR images from prone to supine position for measuring the localized breast density.

US elastography is the most mature and well-documented method for the measurement of tissue stiffness, but it is usually done by using hand-held US probe manually to evaluate abnormal lesions, not the normal tissue. In the present study, by using an automatic US breast volume scanner, we could measure elasticity from multiple ROI’s in a 3D whole breast setting. MR-measured breast density, unlike MD, is three-dimensional and is regarded as an accurate method for measuring volumetric breast density [18, 19, 29]. US-measured breast density has been explored in recent years, but the technical maturity is still in its infant stage [30, 31]. With the volumetric information provided in both US and MRI, we could perform this exploratory study to correlate the measured stiffness and density, in the contralateral normal breast. It is well known that abnormal lesions are stiffer, and as such it may also affect the breast tissue deformation between supine and prone positions; therefore, in this study we only analyzed the normal breast in each subject. Studying the breast tissue density/stiffness change between lesion side and healthy side may be an interesting topic, but it was not the focus of the current study.

The results from this study showed that tissue stiffness measured by US elastography was not correlated with the whole breast density or the local breast density in the US measurement window based on deformed MRI. As age is one of the strong risk factor for breast cancer, in this study we also correlated US-measured breast stiffness and MR-measured breast density with age. It was noted that MR-measured breast density was negatively correlated with age (*r* = − 0.56), but US-measured breast stiffness was not correlated with age (*r* = 0.29). It needs to be emphasized that the current study design recluses any conclusions about risk of breast cancer associated with either density or stiffness. The finding that these two properties of the breast are not correlated does not exclude associated risk of breast cancer. They might interact, or their association with risk might be modified by the presence or absence of other risk factors.

Breast density and tissue stiffness are both known as risk factors for breast cancer, but there was no previous study investigating their correlations using 3D MRI. As both breast density and stiffness are associated with collagen content of the fibroglandular tissue, developing a method that allows investigation of their regional correlation may help to understand whether they are dependent or independent risk factors. Breast MRI is acquired with the subject in the prone position, but the ultrasound is done with the subject in the supine position; therefore, it is difficult to find matched, corresponding, tissue regions. In this study we applied the inversed gravity loaded model to simulate the deformation of the breast from prone to supine position, so the corresponding tissue region on MRI can be estimated based on the US stiffness measurement depth and window.

The breast stiffness of the normal breast tissue was measured by using the SWE, i.e. VTIQ technology. This technique is based on displacement induced ARFI excitation from the transducer, and is quantitative, less examiner-dependent, and with a higher reproducibility [32]. Although the breast stiffness of the normal breast tissue is less studied using SWE technique [17], clinically, SWE has been shown to improve the specificity of ultrasound and allows quantification of stiffness of breast lesions; and it shows that breast cancers are generally stiffer, while benign masses are softer [33–35]. Further, the mean stiffness measured with SWE in ductal carcinoma in situ (DCIS) could predict histological upgrade to invasive cancer [36]. Stiffness of breast cancer was also found to be an independent predictor of lymph node metastasis and thus could provide additional prognostic information for assessment of tumor aggressiveness and staging [37]. In this study the SWE method was applied to measure stiffness of the normal breast tissue for correlation with breast tissue density.

For deformation of MRI from prone to supine positions, we adopted an FEM based strategy. In the prone position the breast was pulled down by the gravity, and the biomechanical simulation was performed to first achieve the zero-gravity state, and then further the inversed gravity state via an iterative algorithm, representing the breast in the supine position. MRI and US were commonly used breast imaging modalities for evaluating lesions, and it was very important to estimate the corresponding location of the lesion in different imaging settings. The matching location was usually done by visual assessment of radiologists based on anatomical landmarks (e.g. quadrants, distance from nipple) shown on US and MRI without the need of breast deformation modeling [38–40]. Some studies analyzed the spatial displacement of the lesion inside the breast using identifiable markers [41], or manual registration methods based on the sensors placed on the surface of breasts [42, 43]. The bio-mechanical simulation method used in this study was mainly developed for surgical navigation, which could avoid bias from the human’s assessments. Previous studies have shown that this method could generate relatively accurate deformed shapes [25, 28]. Nevertheless, it was a simulation and not able to consider elastic properties of all different tissues in the breast; therefore, it could only provide an estimate of the deformed breast. In order to simulate the US images, we used the thickness of the breast as the stopping criterion in the iterative deformation processes. The depth and the window of stiffness measurement on US was then used to determine the corresponding tissue region ROI on MRI for calculation of local breast density used in the correlation analysis.

The FEM method was used in this study to simulate the prone-supine deformation, but it was very difficult to be verified. The deformation of the breast between supine and prone has been a long-standing problem, and so far, there has not been any method published in the literature that can be used to precisely and accurately co-register the breast tissues between these two positions.

In fact, we have started to work on prone-supine deformation on breast MRI more than 10 years ago, and had collected images from the same volunteers with supine and prone positions in one MRI session [44, 45]. With non-rigid co-registration, the breast shapes can be matched perfectly between supine and prone positions; however, it is not possible to verify the accuracy of the results. The registration was done by compressing and stretching the tissues (indicated by the large change in tumor volume), which was not reasonable in reality. Therefore, in this study our goal was only to deform the breast tissue using the gravity model to locate the approximate tissue level, not aiming to find exactly corresponding tissue locations with US ROI using a validated co-registration method.

There are several limitations in this FEM simulation model. Firstly, the material type was determined as Neo-Hookean, which was widely used to model the bio-mechanical materials [25, 28, 46, 47]. However, some previous studies selected Mooney-Rivlin material model [48] in the soft tissue simulation. But in real in vivo scenario, there are many factors that cannot be accurately modeled, e.g. the heterogeneous elastic properties of different tissue components in the breast (collagen, fibrosis, glandular tissue, fat, etc.). Secondly, we fixed the boundaries between breast and chest as boundary conditions and assumed no circumferential stretching between breast and chest. Thirdly, the tension of the skin might play a significant role during the deformation process. In the future work, the circumferential stretching and skin tension need to be considered properly to improve simulation results. Lastly, the breast stiffness measured using US might be affected by the location and breast tissue pattern. In our study, three patients did show very different breast stiffness results (coefficient of variation > 20%) when measured at different locations. Therefore, the goal of the deformation in this study is only to deform the breast to the thickness as in the US measurement setting so the level of the dense tissue ROI measured in US can be located in the deformed MRI.

Only a few studies investigated the association between breast stiffness and normal breast tissue [17, 49–51]. In a study of 132 normal breasts with ROI placed on the local breast tissue, the mean VTIQ values in the breast parenchyma were significantly higher than in the fatty tissue (3.23 m/s ± 0.74 versus 2.5 m/s ± 0.61; *p* < 0.0001) [17]. However, the mean VTIQ values in American College of Radiology (ACR) 1 + 2 versus ACR 3 + 4 density breasts yielded no statistically significant difference [17]. A study showed the association of MD with real time elastography was weak (*r* = 0.44) [49]. The study involved the assessment of B-mode imaging and elastography with regard to their ability to predict 2D MD [49]. This was different from our study of assessing breast density using 3D MRI. In a study examining breast tissue elasticity using US SWE during the menstrual cycle, no significant differences were found in stiffness between glandular and adipose tissues throughout the menstrual cycle; but glandular tissue stiffness was lower in the luteal phase than in the early follicular phase [50]. Tissue stiffness can also be measured by MR elastography. In a study using 3 T MRI, it was found that women with dense breasts had mean stiffness values of 0.96 kPa (center slice) and 0.92 kPa (all slices) while those with entirely fatty breasts had mean stiffness values of 0.85 kPa (center slice) and 0.83 kPa (all slices) (*P* ≤ 0.05) [51]. These results showed that the stiffness is highly dependent on the measurement method and the tissues that are covered. In our study the breast stiffness was measured from echogenic homogeneous fibroglandular tissues by US elastography, so it would have a small dynamic range and not able to show the great difference between dense and fatty tissue as reported in the literature.

Many studies have shown that tissue stiffness is more associated with the alignment and orientation of collagen than the total ECM [52–55]. Histologically, the branching mammary ducts and associated periductal fibrous stroma converge from the peripheral breast tissue towards the nipple. Thus breast tissue close to the nipple area with abundant periductal collagens may express stiffer tissue characteristics than other areas of the breast. We did not find a positive correlation between US-stiffness measured from the dense fibroglandular tissue in the retroareolar region and whole breast percent density, which could be due to regional heterogeneity in the whole breast.

The lack of correlation between US-stiffness and the percent density in the ROI selected for stiffness measurement could be explained with multiple reasons. The measurement of tissue characteristics from the two imaging modalities represents different aspects of the dense tissue. Since the USE measurements were done in dense tissue ROI, the measured results reflected the different tissue properties contributing to the measurement of shear wave speed (elasticity, collagen, and the different distribution of fibroglandular and fat tissue …, etc.). On the other hand, the MRI only measured the averaged volumetric ratio of fibroglandular tissue not related to the tissue elastic properties. As such, the dynamic range may not be sufficient to show a high correlation – by reflecting the amount of dense tissue in both the US and MRI ROI as the dominating factor.

Studies have shown that the local breast stiffness is determined by the interaction and interplay of many factors [56, 57]. The extracellular matrix, which comprises collagens, fibronectin, laminins, polysaccharides, and proteoglycans, plays a key role in the interactions between stroma and epithelium, which are known to influence breast development and the changes in breast structure. Many studies have investigated molecules that mediate the influence of the extracellular matrix on the stiffness of stroma [9]. Therefore, the tissue stiffness was not solely determined by the amount of fibroglandular tissue, and thus not correlated with a simple measurement of local breast density.

## Conclusion

In this study we correlated the US-measured breast stiffness with MR-measured whole breast and local percent density. The stiffness was measured from echogenic homogeneous fibroglandular tissues by US elastography. An automatic, computer algorithm-based, segmentation method was used to segment the whole breast and fibroglandular tissues on MRI. A finite element model was applied to deform the prone MRI to match the supine US images, by using the inversed gravity loaded transformation. Our study didn’t find a positive correlation between US-stiffness measured from the dense fibroglandular tissue in the retroareolar region and whole breast percent density, suggesting that breast stiffness is not solely related to the amount of fibroglandular tissue, and further studies are needed to investigate whether they are dependent or independent cancer risk factors.

## Data Availability

The datasets used and/or analyzed in the current study are available from the corresponding author on reasonable request.

## Abbreviations

ACR: American College of Radiology
ARFI: Acoustic radiation force impulse
DCIS: Ductal carcinoma in situ
ECM: Extracellular matrix
FEM: Finite element model
MD: Mammographic density
MR: Magnetic resonance
MRI: Magnetic resonance imaging
ROI: Region of interest
SWE: Shear-wave elastography
TLED: Total lagrangian explicit dynamic
US: Ultrasound
VTIQ: Virtual touch tissue imaging
